# In Vitro Evaluation of Ferrule Effect and Depth of Post Insertion on Fracture Resistance of Fiber Posts

**DOI:** 10.1155/2012/816481

**Published:** 2012-12-02

**Authors:** R. Schiavetti, G. Sannino

**Affiliations:** Department of Oral Health, University of Rome Tor Vergata, Viale Oxford, 00100 Rome, Italy

## Abstract

*Purpose*. The analysis of the complex model of fiber post and ferrule is given and studied in this paper. A novel approach and a solution to the evaluation of stress of post and core system within the ferrule effect are proposed. *Methods*. Sixty freshly extracted premolars were selected for the study. The following experimental groups were therefore defined (*n* = 10): (1) 5 mm, (2) 7 mm, (3) 9 mm, (4) ferrule-5 mm, (5) ferrule-7 mm, and (6) ferrule-9 mm. Preshaping drills (C) were used to prepare the root canals at 5, 7, and 9 mm in depth. In specimens of groups 3–6 a circumferential collar of tooth structure of 2 mm in height. Fluorocore 2 core build-up material (I) was used for fiber post luting. With the same material, a buildup of 2 mm in height was created. A controlled compressive load (crosshead speed: 0.75 mm/min) was applied by means of a stainless steel stylus (Ø 1 mm) at the coronal end of the post extruding out of the root. *Results*. In all the tests the level of significance was set at *P* < 0.05 . Significantly higher fracture strengths were measured in the presence of a ferrule effect. In groups 1, 2, and 3 (ferrule group), the mean fracture values were, respectively, 163,8 N, 270,9 N, and 254,7 N. These data are higher and statistically significantly different when compared with the three groups 4, 5, and 6 (no-ferrule group), in which the values obtained were, respectively, 40,5 N, 41,7 N, and 44,9 N. *Conclusion*. The ferrule effect in the endodontically treated teeth positively affects the fracture strength of the fiber post. Conversely, post depth insertion did not affect the resistance to fracture.

## 1. Introduction

A persistent problem in clinical dentistry is represented by the risk fracture of endodontically treated teeth [1]. These teeth are considered to be less resistance, because of the loss of tooth structure during conservative access cavity preparation. The influence of subsequent canal instrumentation and obturation leads to a reduction in the resistance to fracture [2, 3]. To restore these teeth, posts are often required in order to provide anchorage for the core-forming material and coronoradicular stabilization [4, 5]. Cast posts and cores have been used for this purpose for many years, while more recently fiber posts showed to represent a valid alternative. The clinical success of fiber post restorations is mainly related to their biomechanical properties that, being close to those of dentin, reduce stress transmission to the roots [6–9]. The potential of fiber posts to reduce the incidence of nonretrievable root fractures in comparison with cast posts was confirmed in several studies [10–12]. Among the several parameters influencing the success of a post-based rehabilitation, preservation of coronal dental tissue and, particularly, the presence of a ferrule effect have been advocated as favorable conditions to decrease stress transmission to the root [13]. Sorensen and Engelman [14] described the ferrule as the coronal-dentinal extension of the tooth structure occlusal to the shoulder preparation. The ferrule effect in association with cast post and cores has been studied by many investigators [15–17]. Conversely, little information is available if the ferrule is of additional value in providing reinforcement in teeth restored with prefabricated post and composite cores, and the advantages coming from the presence of ferrule in prefabricated post and core are questioned by Al-Hazaimeh and Gutteridge [18].

The main task of this in vitro study is to evaluate the effect of ferrule preparation on fracture resistance of fiber post, as a function of the presence/absence of a ferrule and as a function of the depth of insertion of the fiber posts. 

The formulated null hypothesis was that neither depth of post insertion nor the presence of a 2 mm high ferrule had a significant influence on fracture resistance of a fiber post- retained restoration.

## 2. Material and Methods

Sixty freshly extracted premolars were selected for the study. Teeth had to be free of cracks, caries, and fractures and were stored at room temperature in saline solution before testing. The anatomic crowns of all teeth were sectioned perpendicularly to the tooth long axis at the cement-enamel junction (CEJ). Roots were endodontically treated using the “step-back” technique [19] to a number 70 size file (A) (see Table 2) and irrigated with 2.5% sodium hypochlorite.

**Figure 1:**
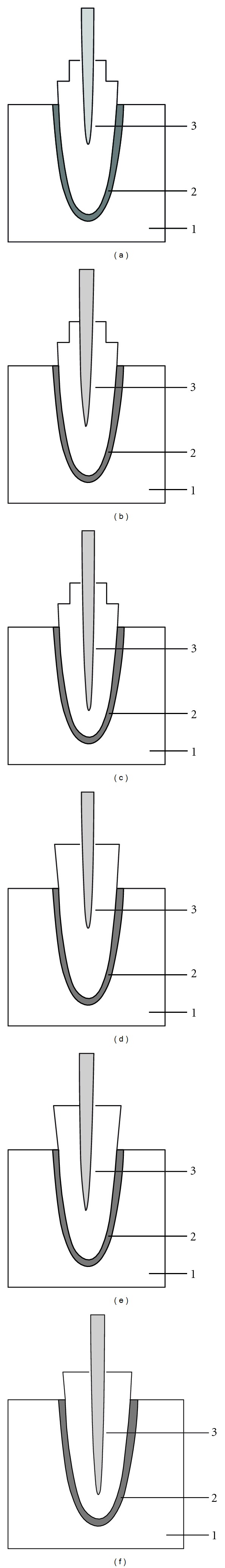
Experimental groups with different post depth (5, 7, and 9 mm) and postspace with (groups a, b, c) and without (groups d, e, f) a ferrule effect.

**Figure 2:**
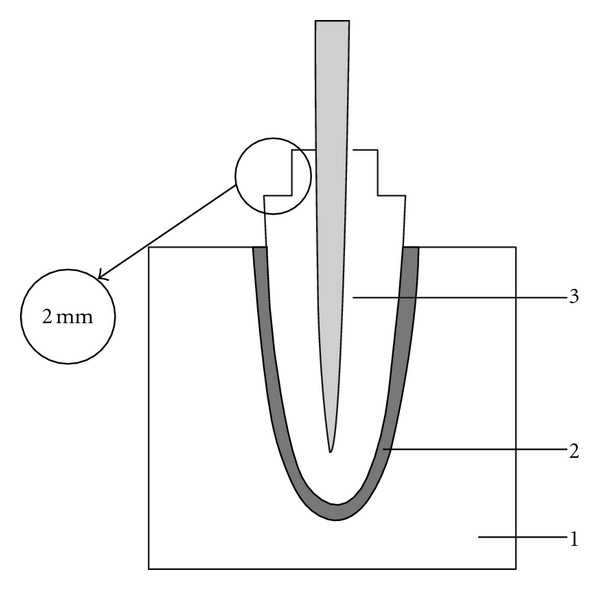
Ferrule effect. A circumferential collar of tooth structure at least 2 mm in height was preserved at the gingival aspect of the preparation.

**Figure 3:**
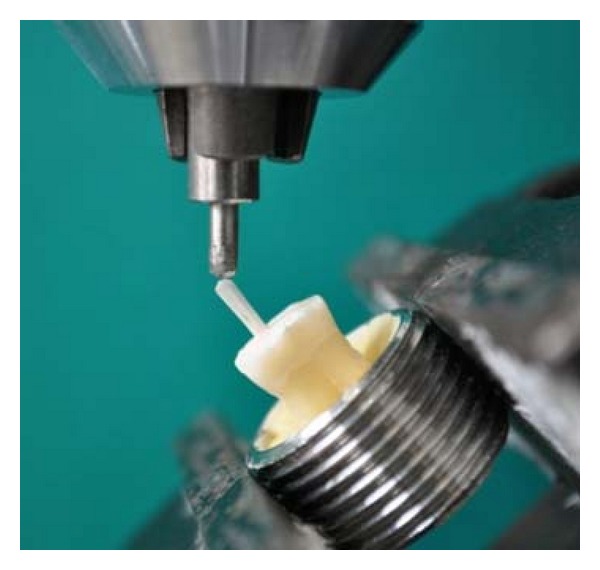
Example of a sample mounted on the loading machine and prepared for the fracture test. The tooth is oriented such as the load applied by means of the metallic stylus would have a 45-degree direction.

Each canal was obturated using the lateral condensation technique with gutta-percha points (B) and the resin sealer AH Plus Jet (C) (see Table 2). The endodontic access cavities were temporarily filled with a glass ionomer cement (D) (Fuji II, GC corp, Tokyo, Japan). After 24 hours, the coronal seal was removed by means of 240-grit abrasive SiC papers under water cooling. Roots were randomly divided into six experimental groups that differed for the depth of the prepared post space and for the presence or absence of a ferrule effect. The following experimental groups were therefore defined (*n* = 10): (1) 5 mm (Figure 1(a)); (2) 7 mm (Figure 1(b)); (3) 9 mm (Figure 1(c)); (4) ferrule-5 mm (Figure 1(d)); (5) ferrule-7 mm (Figure 1(e)); (6) ferrule-9 mm (Figure 1(f)). Preshaping drills (C) were used to prepare the root canals at 5, 7, and 9 mm in depth. After preparation, it was checked that a 3-mm long gutta-percha apical seal. In specimens of groups 3–6 a circumferential collar of tooth structure of 2 mm in height and 3 mm in width was realized with a diamond bur (Figure 2). 

Translucent quartz fiber posts (E) consisting of unidirectional, pretensed fibers bound in a translucent epoxy resin matrix, were used. Each post was tried in into the root canal, and the portion of the post extruding out the root was cut to a standardized length of 4.8 [20]. Prior to cementation, a prehydrolyzed silane coupling agent (F) was applied with a microbrush on the post surface for 30 s. The light cured, self-priming adhesive Prime and Bond NT (G) was applied into the root canal with a microbrush for 20 s and gently air-dried. The excess was removed using paper points. The bonding agent was polymerized with a conventional quartz-tungsten-halogen light (750 mW/cm^2^) (H). Fluorocore 2 core build-up material (I) was used for fiber post luting. Base and catalyst (1 : 1) were mixed for 30 s, then the material was applied on the post. The post was seated immediately into the canal and sustained under finger pressure. With the same material, a buildup of 2 mm in height was created. After the first 7-minute autocure period, the material was light-cured for 40 s. After curing, the specimens were prepared as for a prosthetic crown, with a circumferential chamfer reduction of 1,5 mm of maximum thickness, using a chamfer bur of 2 mm in diameter (M). After post cementation, each root was embedded in a self-polymerizing acrylic resin (J) for half of the root length, with the long axis sloped at a 45-degree angle to the base of the resin block. During this procedure, specimens were continuously irrigated with water to avoid overheating due to resin polymerization. Before performing the mechanical test, samples were stored for 24 hours at 37°C and 100% relative humidity.

Each sample was then mounted on a universal testing machine (K). A controlled compressive load (cross-head speed: 0.75 mm/min) was applied by means of a stainless steel stylus (Ø 1 mm) at the coronal end of the post extruding out of the root (Figure 3). A software (L) connected to the loading machine recorded the load at failure of the post- retained restoration measured in Newton (N).

## 3. Results

Descriptive statistics of fracture strength data are reported in Table 1, along with the significance of between-group differences. As the distribution of fracture strengths was not normal according to the Kolmogorov-Smirnov test, the use of the Two-Way Analysis of Variance to assess the influence of depth, ferrule effect, and between-factor interaction was precluded. Therefore, the Kruskal-Wallis One-Way Analysis of Variance was applied with strength as the dependent variable and experimental group as factor. Consequently, the Dunn's multiple range test was used for post hoc comparisons. In all the tests the level of significance was set at *P* < 0.05. Significantly higher fracture strengths were measured in the presence of a ferrule effect. Neither in the presence or in the absence of a ferrule effect had depth of post insertion a significant influence on fracture strength, as no statistically significant differences emerged either among groups 1–3 or among groups 4–6. 

The results obtained from this in vitro study showed a correlation between the presence of the ferrule and increased resistance to fracture. In groups 1, 2, and 3 (with ferrule), the mean fracture values were, respectively, 163,8 N, 270,9 N and 254,7 N. These data are higher and statistically significantly different when compared with the three groups 4, 5, and 6, without ferrule effect, in which the values obtained were, respectively, 40,5 N, 41,7 N, and 44,9 N. 

The depth of post insertion did not show to be a parameter affecting the results. In fact, no statistically significant differences were found between groups 1, 2, and 3 as well as between groups 4, 5, and 6.

## 4. Discussion

Since in the presence of a ferrule, significantly higher fracture strengths were measured, the null hypothesis has to be rejected.

Several factors determine the performances and the success of a rehabilitation clinic in time: types, design, and lengths of post, bonding capacity [21], and ferrule. Large variations exist in regard to the physical and fatigue resistance of resin-fiber posts [22]. The static or dynamic behavior of resin-fiber posts depends on the composition (fiber type and density) as well as the fabrication process and, in particular, the quality of the resin-fiber interface. In an in vitro study examining physical properties of various posts, it was concluded that the ideal post design comprises a cylindrical coronal portion and a conical apical portion [23]. Much discussed is still the ideal post length, if one part provides greater stability to prosthetic rehabilitation at the same time involves removal of dentin [24] and more because of the existing limitations of adhesive procedures within the root canal [25–27]. It has been demonstrated that the loss of tooth vitality is not accompanied by significant change in tissue moisture or collagen structure [28–30]. The most important changes in tooth biomechanics are attributed to the loss of tissue either at radicular [2, 31] or coronal [31–34] levels, pointing out the importance of a highly conservative approach during endodontic and restorative procedures. The significance of remaining cervical tissue, known as the ferrule, was also well documented [13, 35]. The incorporation of a ferrule is an important factor of tooth preparation when using a post-supported rehabilitation technique [36–38]. The effectiveness of the ferrule has been evaluated with several laboratory tests as fracture resistance, such as [39] impact [40], fatigue [41], and photoelastic analysis [42]. According to these studies the ferrule presence showed values of resistance to fracture much higher and statistically significant differences in groups 1, 2, and 3 than no-ferrule groups (groups 4, 5, 6). Concerning the length of the ferrule, some studies have reported that a tooth should have a minimum amount (2 mm) of coronal structure above the cement-enamel junction (CEJ) to achieve a proper resistance [43, 44].

The results of the present study, in which to assess the mean fracture for each group the force was applied directly on the post head, in order to exclude other variables, have confirmed these observations.

About post insertion depth, it is known that with cast post and core system the post length was an important variable, because reducing post space can permit to save tooth structure positively affecting the tooth fracture resistance. Some authors [45] in a recent study designed to obtain a biofaithful model of the maxillary incisor system and to assess the effect of glass fiber post lengths using Finite Element Analysis showed that the overall system's strain pattern did not appear to be influenced by post length. This could lead to the conclusion that a post inserted more deeply could be more effective in a fiber post-supported rehabilitation, as the length of the post insertion has a significant effect on retention; the more apically the post is placed in the root, the more retentive is the system [46–48]. This consideration should not be overestimated in clinical practice. The adaptation of the canal shape to the post [49] and the overall length of the root should be in fact taken into consideration, because it has been reported that a residual apical filling of less than 3 mm may result in an unpredictable seal [50, 51]. 

From the results of the present study, a tendency of the more deeply inserted post to have higher values of resistance to fracture could be anyway observed, particularly in the no-ferrule groups. This might be connected with the use of tapered post, considering that a post inserted more deeply has a wider diameter at the breaking point. The use of a cylindrical shaped post could have minimized this differences, and this could be considered as a limit of the present study, even if Lang et al. [52] showed that if an excessive amount of tooth structure is removed and the natural geometry of the root canal is altered, this will have a destabilizing effect on root-filled teeth. For this reason in clinical practice the use of cylindrical-shaped post have been progressively abandoned and replaced with tapered post.

As general consideration, it should be noted that this in vitro study does not reproduce the exact clinical conditions, where lateral forces should be considered as well as axial forces and fatigue loading, ageing processes, alternate thermal stress, mechanical stress, wear, and water storage. In this in vitro study, in fact, lateral forces were applied with a 45° angle between the post and the loading tip. Moreover, stress applied to the teeth and dental restorations is generally low and repetitive rather than being isolated and loading. However, because of a linear relationship between fatigue and static loading, the compressive static test also gives valuable information concerning load-bearing capacity [53, 54]. Based on this statement, the results of this in vitro study showed that the ferrule effect positively affects the resistance to fracture of endodontically treated teeth restored with fiber posts. Conversely, post depth of insertion did not affect the resistance to fracture. 

## 5. Conclusion

Within the limitation of this in vitro study, the statistical results showed that the ferrule effect in the endodontically treated teeth positively affects the fracture strength of the fiber post. Conversely, post depth insertion did not affect the resistance to fracture. It could be advisable in the rehabilitation of endodontically treated teeth preserve radicular tissue, reducing the postspace preparation, in order to improve the fracture strength of the post with a ferrule length of at least 2 mm.

## Figures and Tables

**Table 1:** Descriptive statistics of fiber post fracture strength data with the significance of between-group differences.

Number group	Name group	*N*	Mean	Std. Deviation	Median	25%–75%	Significance *P* < 0.05
1	Ferrule-5 mm	10	163,8	72,5	142,9	132,7–181,1	AB
2	Ferrule-7 mm	10	270,9	105,6	244,9	215,2–350,3	A
3	Ferrule-9 mm	10	254,7	79,1	235,4	193,4–305,6	A
4	No ferrule-5 mm	10	40,5	3,1	40,2	38,4–44,2	C
5	No ferrule-7 mm	10	41,7	5,3	43	36,8–46,2	C
6	No ferrule-9 mm	10	44,9	6,7	44,5	40,5–51,7	BC

**Table 2:** Classification of instruments used for collecting and measuring data during the tests.

Class	Type
(A)	Flex R File, Union Broach, York, PA, USA
(B)	Dentsply, Maillefer, Tulsa, OK, USA
(C)	DeTrey, Konstanz, Germany
(D)	Fuji II, Gc corp, Tokyo, Japan
(E)	ENDO LIGHT-POST number 3 Bisco, Schaumburg, IL, USA
(F)	Monobond S Ivoclar Vivadent, Schaan, Liechtenstein
(G)	Prime and Bond NT Dentsply DeTrey, Konstanz, Germany
(H)	Optilux 401 Kerr, Danbury, USA
(I)	Fluorocore 2 Dentsply DeTrey, Konstanz, Germany
(J)	ProBase Cold Ivoclar Vivadent, Schaan Fürstentum, Liechtenstein
(K)	Instron Corp, Canton, MA, USA
(L)	Digimax Plus Controls srl, Cernusco s/n, Italy
